# Letters of Guy Patin

**Published:** 1847-07-01

**Authors:** 


Lettres de Gui Patin. Nouvelle Edition augmentée de Lettres Inédites, et precedée d'une Notice Biographique. Par *J. H. Reveillé-Parise,* Docteur en Medecine, &c.

Letters of Guy Patin. A new Edition, augmented by Inedited Letters, and preceded by a Biographical Notice. By *J. H. Reveillé-Parise,* M.D. Three volumes, 8vo, pp. 1974. Paris, 1846.

Guy Patin may claim a foremost place in a department of literature—the difficult art of letter-writing—in which his countrymen have attained an acknowledged superiority; and the only regret we feel at the conclusion of our perusal of the portion of his voluminous correspondence here presented to us by M. Parise, arises from the fact of its termination having been reached, and our conviction that, take what pains we may, we can only present our readers with a very inadequate idea of its interesting characteristics. Translation may be undertaken by the practised hand in the case of didactic or narrative literature with the assurance of an accurate, and even an elegant, execution; but when vivid pictures of men and manners, dashed off in a few lines of happy but idiomatic phraseology, are attempted to be thus transferred, they are apt to lose all their original brilliancy, and even become distorted by the different refracting power of the medium of transmission. Thus it is that attempts at the adequate rendering of the various celebrated French letter-writers have always proved marked failures—washy pointless productions being substituted for the elegance and vivacity of the originals. We may, however, indicate to our readers what they will find by referring themselves to these volumes, and can assure them that the various scenes therein depicted by the ever-active writer are endowed with a life-like energy that at once transports us to the scene of action, mingles us with the throng of events, forcibly impresses the imagination, and conveys a more accurate idea of the manners of the epoch than volume upon volume of history written for the express purpose could impart.

Guy Patin was born, in 1601, in the province of Picardy. His father was an Advocate, but, being tempted by the inducements held out by the Seigneur of his village, he left Paris, where he was endeavouring to acquire practice, and established himself as a kind of steward or superintendent of that gentleman's estate. The promises which had been made him not having been fulfilled, his circumstances always continued very limited. Guy often refers to the excellent moral character his father bore with becoming pride. "My father," says he, "was a good man if ever there was one. If every one was like him we should have little need of lawyers. He came to Paris annually on his master's business, and his credit was always excellent. I found, on my arrival there, numbers of friends whom I knew nothing of, but who loaded me with kindness on his account, which has made me many a time deplore his loss more and more." His father gave him a good preliminary classical education, and as soon as he had left college, some of the nobility of the district, who had become indebted to his father and were desirous of paying him by what cost them nothing, offered him a living for his son: but Guy, with a spirit which seemed to foreshadow his future energetic opinions upon the subject, "flatly refused it, protesting absolutely I would never become a priest; blessed be God for so impressing my mind at so early an age. My father recognising in this refusal something good and ingenuous, was in no-wise irritated by it; but my mother continued exasperated against me for more than five years." Referring to the subject some forty years after, in a letter to a friend, he says, "I have oftentimes praised God that he never made me either a woman, a priest, a Turk, or a Jew."

The anger of his mother caused him to leave his home, and he came to Paris to continue his studies. What his resources were he mentions not, but Bayle tells us he supported himself as a corrector of the press, Drelincourt, Professor of Medicine at Leyden, having taught him this. "We must not be surprised," says M. Parise, "at Patin choosing such a profession to gain a livelihood. At this period, as well as in the preceding century, it was the occupation of many distinguished men of letters, and especially of Erasmus and his friend Budæus. It sometimes happened that a philosopher printed in the morning that which he had written the night before." While so occupied, however, he fell in with the celebrated Riolan, who induced him to study medicine, and in 1627 he received his Doctor's degree, which effectually appeased the ire of his mother. He soon acquired reputation and practice, and having married a rich wife was henceforth placed at his ease in regard to pecuniary concerns. His practice also rapidly augmented, and Riolan, having quarrelled with his son, obtained for Patin the reversion of the professor's chair he held in the college of France, to which he did not succeed however until 1654.

He was not the man to vegetate in wealthy ease, or to pursue his profession as a mere lucrative calling. On the contrary, his activity was prodigious and usefully directed in the main. As was the custom of the period, he entered into a voluminous epistolary correspondence with the learned in various parts of Europe. He accumulated a large library of valuable books, which he not only read but commented upon with great critical acumen. He delivered lectures to crowded auditories, and joined in or presided over the disputation of theses with a zest and erudition which even the learning of that epoch exhibited few examples of. Thoroughly imbued with the conviction of the dignity of the medical art, and of the superiority of the scholastic and Galenical mode of acquiring and practising it, he was in continual war with the charlatans, the chemists, the apothecaries, the barber-surgeons, and the hosts of unqualified practitioners of the day. A warm sympathizer in the cruel sufferings the masses of his countrymen were subjected to by the oppressions of the priesthood and noblesse, and by the corruption which pervaded all society from the Court downwards, many of his letters are occupied in stigmatizing the authors and causes of these, evincing an amount of information which the most active intercourse with general society could alone furnish him with. Notwithstanding his extensive town-practice, and his being frequently called away from Paris (journies he always bitterly complains of as tearing him away from his beloved books), he could still find time to direct his son's or his pupils' studies, to expound some passage of Galen or Fernel to them, and to endeavour to impress upon them the elevated objects and the responsibilities of the profession they had chosen.

He was a man of strong feelings and prejudices, and the manners of his times permitted the giving currency to these in terms which the more subdued temper of our own has properly discountenanced. A withering sarcasm, a great facility of expression, whether in Latin or French, an utter carelessness of wounding the feelings of those attacked, must have rendered him indeed a formidable enemy; and nothing can be happier than many of his sarcastic allusions and cutting epigrams. But if he was a fierce opponent, no mean or personal considerations prompted his enmity. He neither manifests anxiety for lucre or jealousy at the legitimate success of brother-practitioners. On the contrary, he refused appointments which would have torn him from his books and other associations, and mentions with eager delight the instances of good fortune which befel his friends and colleagues. The bitter reviler of Cardinal Mazarin and the Jesuits, the harsh antagonist of the lower grades of the profession in their laudable efforts to raise their position, the cordial hater of the chemists and charlatans of the day, expresses with admirable pathos his sympathy for the miseries under which his country groaned, was a doating though not a foolish father, and could drop a tear when a beloved colleague or favourite author left this mortal scene. His friendships once formed seemed immutable by time and circumstances, and so acutely did he feel the death of Grotius that he at once fell ill, and was not enabled to resume his duties for several days.

But the most admirable trait in Guy Patin's character was his noble frankness and love of truth, and detestation of hypocrisy and meanness of every description. That several of his views and prejudices were unfounded and exaggerated, events have proved; but no doubt as to the purity of his motives, the uprightness of his conduct, will ever be entertained. It will be believed that qualities such as we have mentioned, procured for him an abundant supply of enemies. He was well aware of this, and, full of courage and confidence in his cause, was ever ready for the combat, and never contented with taking the mere defensive. But so likewise the goodness of his heart, his upright character, his active disposition, learned eloquence, and great practical knowledge ensured him numbers of attached friends and a vast reputation, which continued long after his decease. Unfortunately, beyond a few theses and ephemeral publications, he issued nothing from the press, so that the opinion posterity will form of him must rest upon his Letters, several collections of which, addressed to medical men and literati, have been published, but none having the least pretension to completeness or accuracy, until the present one brought out under the able superintendence of M. Reveillé-Parise. Guy Patin continued his active career until 1672, when he died, having undergone much vexation in his latter years from the ingratitude of his elder son, the exile for reasons he never could divine of his younger and favorite one, and from the rapid progress which the chemical practitioners made.

To a few of the multifarious subjects embraced in his correspondence we may now direct attention, and first to the writer's ideas upon the practice of physic. These were eminently scholastic. Galen is his deity, and Fernel, as the ablest expounder of Galen, his arch-priest. By this test must every doctrine be examined and every practice measured. Although, certainly, the Galenical views and his own sound sense sometimes conducted our author to modes of treatment which would be approved of even in our own day, yet the manifest advance we have made will be at once seen by those who consult his book. He made little or no use of drugs, save purgatives, and sternly prohibited the use of opium, cinchona, or antimony, then beginning to be introduced, under any circumstances whatever. Although confiding much in Nature's powers, he by no means stood by as an idle spectator, for, if he declined interfering with her operations by means of the materia medica, he was by no means chary in the employment of the lancet. Perhaps he is the most daring phlebotomist on record, glorying in the victories he achieved, and regarding those who objected to his practice as rash with the greatest contempt. Here are a few of the innumerable passages in his letters illustrating this point.

"He died in the 48th year of his age of a continuous fever of twelve days duration, for which only two small bleedings were performed. So much for bleeding in Italy! About that time my son was also very ill; but I rescued him from a bad condition of continued fever into which he had unfortunately fallen, *quia adolescentuli semper stulté agunt,* by means of *twenty* free bleedings from the arms and feet, with at least a dozen good purgations with cassia, senna, and syrup of pale roses, without ever using your bezoards, juleps, cordials, or confections of hyacinth or alkermes; and yet God has preserved him to me, in such a manner that he has not lost even a single lecture. * * * * * Our Paris people usually take but little exercise, drink and eat a great deal, and become very plethoric; and in this condition they are never relieved of any disease that may attack them, if bleeding is not powerfully and copiously first resorted to; and yet, if it is not an acute disease, the effects are not so soon visible as from purgation. About the year 1633, M. Cousinot, now first physician of the King, was attacked by a severe and violent rheumatism, for which he was bled *sixty-four* times in eight months, by order of his father and M. Bouvard. After being so often bled, they began to purge him, by which he was much relieved, and at last cured. The idiots who know nothing of our art, imagine that to purge is sufficient: but they are much mistaken; for if bleeding has not preceded, to repress the impetuosity of the wandering humour, to empty the large vessels, and correct the intemperature of the liver, which produces this serosity, purging could not prove useful. I have heard him say himself that the bleeding would have alone cured him, and that without it the purging would have been of no avail. Formerly, I was called to a young gentleman of this place seven years of age, who fell ill of an acute pleurisy. His guardian disliked bleeding very much, and I could only oppose his hatred of it by a piece of good advice, that he should call in two of our seniors, MM. Seguin and Cousinot. The lad was bled *thirteen* times and cured as if by a charm, so that the guardian himself was converted.
* * * * * * * *
Since I last wrote to you, I have been seized with so horrible a cold that I have been obliged to quit every thing and go to bed, where I have been bled *seven* times. God be praised I am now rid of it, and only want strength. * * * * * * To prevent the marks of the small-pox, we employ here tepid almond oil. But I think the best remedy is bold venesection from the commencement of the disease *ad contemporandum fervorem et extinguendam acrimoniam sanguinis exuberantis ex utrâque basilica,* and steeping, during the first twelve days, the eyes and the face *ex aqua optima tepida; qualem hic habemus sequanicam,* so as to procure the evaporation of his malignant humour imprisoned under the skin. * * * * * *
I have just been reading something in your Sennertus; I am quite in a rage: first, on account of the great number of faults there is, and secondly, because this good man was new at, and understood little of, practice. He knows nothing about bleeding old persons and children; for see what he says at p. 616 of his first volume. This miserable example excites my pity. I think the poor man has scarcely ever seen any patients, and that *nullus fuit in praxi saltem admodum indiguit Delio Natatore*. If we acted thus at Paris all our patients would quickly die. We cure patients more than eighty years old by bleeding, and bleed, just as successfully and without any ill effects, children two or three months old. I could point out at least two hundred persons living here who were bled in their infancy. I think the patients in Germany are much to be pitied with such physicians, who have indeed only the names of the qualities they bear, and who, understanding nothing of remedies or method, seek for the secrets of chemistry in Paracelsus and Crollius, who were never physicians at all. A day does not pass in Paris in which we do not bleed several infants at the breast and septuagenarians *qui singuli feliciter inde convalescunt*. There is not a woman in Paris who does not believe in its efficacy, and who would refuse to allow her infant to be bled for the fever of small-pox or measles, convulsions, or teething, so convinced have they become by experience."

Patin explains in many places that, it is not blood which is drawn from the veins on these numerous occasions, but mere " matter," " sanies," or " corrupted humours." Energetic, however, as he was in wielding the lancet and in the subsequent employment of purgatives, the various active remedies then becoming introduced into practice were met with his absolute prohibition and utter aversion—the very name of " specific" exciting his indignation and contempt. The Chemists, following in the footsteps of Basil Valentine and Paracelsus, employed the most energetic endeavours to procure the admission of some of the various mineral preparations, especially of mercury and antimony, into the materia medica; but to this the Galenical physicians opposed the stoutest resistance, and were long successful in their opposition, especially in France. In our own country, Sir Theodore Mayerne, alike skilled in Galenical lore and in chemical knowledge, contributed much to soften animosities and prepare the way for innovations; but, in Paris, the bitterest contentions prevailed, and no form of speech was deemed too harsh for employment, and no imputation too odious for assertion. Guy Patin repeatedly refers with pride to two solemn decrees made by the Faculty of Medecine in 1566 and 1615 against *Antimony* as a virulent poison, decrees which were afterwards sanctioned by the Parliament; and although, towards the latter part of his career, great numbers of the doctors of the Faculty had gone over to the chemists, he could never persuade himself that this was the case, until at last the opponents of the Galenists were strong enough to obtain, in 1666, a formal reversal of the obnoxious decrees. Nothing could convert Patin, his disbelief in the virtues of the medicine being as complete, and his diatribes against those who employed it as virulent from first to last. There can be no doubt that the use of antimony, at first administered by the hands of charlatans and in considerable doses, was frequently followed by the evil effects he represents as always attending its employment; and even in the hands of those of the regular physicians who employed it, it seems frequently to have induced death. Any one of the Faculty venturing to recommend, or even sanction, its administration was at once degraded in Patin's eyes to the level of the lowest charlatan, and stigmatized accordingly. Speaking of one of these named Guénaut, Patin says:

"Antimony is here much decried. Guénaut's third daughter was buried on the 18th. She died in her confinement of her second child. Her hangman of a father is so wicked, that in this last illness of hers he ordered her to take the emetic wine six times. I think the man is mad, or is possessed of the devil. Most families are complaining of this poison, and yet Guénaut and some others are only the more determined to give it, declaring in mockery, 'Come, it's not so bad as they say: if it's not good for those who take it, it is at least so for their heirs.' Thus do they play with the lives of men, and yet impunity prevails everywhere. May God protect us from such drugs and such physicians!"

In other places he says :—

"I send you the epigram M. Augier has written upon the "Triumph of Antimony." It is approved of by everybody as antimony is detested by all honest persons, having only on its side charlatans, empirics, apothecaries and such like rabble. * * * * Observe how Messieurs the Antimonians play with the lives of men, and impudently send their poor patients into the other world with their poison, under the pretence of having particular secret remedies, the very terms of charlatans, *a quibus decipiuntur idiotæ tam togati quam tunicati.* The great desire to be deceived, and the little cannot help being so. * * * * The ministers and Mazarin are the demons of France, the Turk of Christianity. The chemists, apothecaries, and charlatans are, in their kind, the demons of the human race. The pretended demon of the infernal regions kills not so many as does this chemical demon and chemical poison. * * * * * * The wife of Meyssonier is dead then from the emetic wine! This poison seems to be doing its work at Lyons as well as Paris. Some of your doctors too have given it to their wives who will never take it again; seeing that, by the grace of God, they are all dead, and some of them here have taken younger ones in their places. Guénaut has had his family in mourning three times, and has killed so many in different places that he now nowhere dares propose its administration."

But, alas! poor Patin. Antimony pursued its triumphant career, in spite of no one, as you say, daring to give it, and the young King, Louis XIV. falling ill at Calais, it was administered to him—and with success. An empiric was brought expressly from Abbeville, who, seating himself on the King's bed, exclaimed, "This lad is very ill, but die he will not," and in a short time he was completely cured. Patin utterly denies that antimony exerted any agency here, and then consoles himself and his friends with retailing the epigrams made on the great delivering themselves into the hands of quacks and imposters. Even hexameters and pentameters stigmatizing *Stibii noxæ vindiciæ* were free circulated. The chemists were by no means disposed to remain idle, and met their opponents upon their own field, after the example of Paracelsus, with pen in hand, and nothing can exceed the indignation of Patin and his brethren at the treatment they received in the Legend of Antimony and Antimony Triumphant. Even persons unconnected with medicine mingled in the fray, and a monk named Carneau composed a burlesque poem upon the war waging between the rival practitioners, which M. Parise thinks may have suggested to Garth his, in every way superior one, entitled the Dispensary. Here is Patin's amusing version of the king's illness.

"The King is a well-made, strong, and tall prince, twenty years of age, who has never drank or injured his frame by any kind of debauchery. His disease was but an excess of heat from having ridden too long in a burning sun, which, according to Galen, is one of the most powerful external causes of disease; assisted by the infected air of the marshy districts in which the army is encamped. It was a putrid continued fever, which only required bleeding and a refreshing diet, with slight purgatives. * * * * * Three purgative apozemes, composed of cassia and senna, were prepared. The Cardinal inquired if there was anything unusual to be put into them. Esprit, physician to the Duke of Anjou, said some of the emetic wine might be added. Pretty politics these of our times! The physician of the next heir and immediate successor to the Crown called into consultation, and daring to prescribe his stibial poison. If his advice had been followed and the king had died, his master would have become king, and he the king's first physician. *Non sic erat in principio.* Formerly the physicians of the princes of the blood were never called in to a sick king, for very good political reasons; but now-a-days everything is reversed. Guénaut stated that the emetic was in no-wise to be feared if only a little were used, and Mazarin therefore directed it to be given, an ounce of the wine being placed in three doses. The king took one of these, which in two hours began to act, and in the course of the day he went twenty-two times to stool, which fatigued him much. In the evening, his fever was much increased; he passed a very bad night, and early in the morning they were obliged to bleed him, and regretted having given the antimony, for if matters had become still worse from it they would not have failed to have suffered. The king was again bled twice, and then re-purged, after which he felt better. So foolish is it then to say that the emetic wine saved him, since he took as little as could be; and no other medicine would he take until they swore to him it contained none of it, so much did he detest it. What saved the king was his innocence, his age and strength, nine good bleedings, and the prayers of *worthy people like us*, and especially of the courtiers and officers, who would have been terribly afflicted at his death, particularly Mazarin." P. 91.

We see, by the above extracts, Patin, as indeed was the custom at the epoch in which he flourished, dealt in the most odious imputations. His phraseology was not always very choice, and thus the terms impostors, charlatans, stibial torturers and executioners, &c. are of constant occurrence. "Chemistry," he says in one letter, "is the false coinage of our art," and on several occasions he relates the punishment of persons engaged in chemical pursuits as coiners. Doubtless at this period, when the philosopher's stone was still an object of alchemical research, many availed themselves of the cloak this offered them for their designs. After the decree for reversing the edict against antimony was passed, Patin writes:

"These gentlemen declare a poison is not a poison in the hands of the physician. They speak against their own experience, for most of them have killed their wives, their children, or their friends with it; and they speak well of the drug in order to curry favor with the apothecaries, although they do not dare to taste a drop of it themselves. I console myself; for there must be heresies in order that good men may be proved; but it has never been my humour to worship the golden calf, or to consider fortune as a goddess. God preserve me from doing so in future. I am content with the mediocrity of mine, Peace and little. When the wind changes, all these champions of antimony will be dissipated like the smoke of their own furnaces. *Ipsi peribunt; dii meliora piis—Vale.*"

It was not only heresies among the learned Faculty that Patin vainly endeavoured to oppose, but likewise the encroachments of the *Apothecaries* and the *Barber-Surgeons*, against whom he is quite as severe as against the veriest charlatans. His especial aversion was the poor apothecary, whom he considered an useless and expensive appendage, covering with well-deserved ridicule the complex and absurd formulæ then so much in vogue. His indignation was also excited by the patronage afforded the apothecaries by certain of the Faculty for their mutual advantage, at the expense of the patient. The apothecary was evidently then beginning to be much employed, and developing into the general practitioner; and Patin and his colleagues, as well as the practitioners of our own country, would have been better employed in aiding, rather than obstructing, his attempts at placing his position upon a sounder basis, by means of an improved education.

So violent were the diatribes of Patin, that the Apothecaries brought an action against him for libel; but this he defended without the help of any advocate, to the great discomfiture of his assailants. "This trial," he writes, "has brought me nothing but honour, and made my thesis well known, for every body is asking for it." He endeavoured to countermine them in another way by publishing a small work, entitled the "Charitable Physician," which met with great success, and instructed the public in the mode of preparing the few purgative potions and enemata he employed. Indeed, his pharmacopœia seems to have been limited to manna, senna, rhubarb, cassia, and the syrup of pale roses, by the help of which, bleeding, and an appropriate regimen, he maintains all curable diseases were to be cured. He declares that the Faculty would have rid Paris in this way of the whole tribe, had not some of its members found their interest in conniving with the apothecaries. Addressing a friend at Lyons, he writes:

"You have come to an agreement then with that pest of medicine the apothecaries. They deserved no such favour from their masters, upon whom they should absolutely depend. If you wish to prevent their presuming and encroaching upon you, remind them that the 'Charitable Physician' has well-nigh ruined those of Paris. Make them understand that the grocers sell rhubarb, senna, and syrup of pale roses, with which remedies we do without them, and have rendered them so ridiculous that they are no longer seen in families, and have somewhat too much leisure now to look after their shops. God be praised we hear nothing now of bezoards, cordials in the small-pox, cordial julep, and pearls in all kinds of diseases. The people are undeceived, and are much obliged to several of our faculty for having delivered them from the tyranny of these Arabic cooks. Those who used to complain of the great expence and excessive charges of the apothecaries were the first to become undeceived; and it is observable that, whereas heretofore we were constantly seeing Apothecaries bills brought before the law-courts to be taxed by the physicians appointed for the purpose, we see this now no longer. * * * The people of Paris have become so accustomed to the saving, that when they become ill they no longer seek for the apothecary but send at once to the physician; and although, owing to the badness of the times, there are several patients who do not pay very well, yet at least we have the advantage of being the first called, and we do not see at their houses that litter of juleps, apozemes, powders, opiates, and cordials, which for the most part only served to prolong the diseases, and heat and disgust the patient, besides proving very expensive to him."

Subsequently he writes as if this defeat, as in truth it was, were rather desired than accomplished.

"So little do I care for this sort of persons, that they excite neither my envy or pity, and never will I, by God's help, deceive a poor patient to gratify them. Strange it is indeed that they so easily find certain persons in our profession, who, abandoning their honour and conscience, undertake the defence of so deplorable and unfortunate a cause. We ought to hate them as a pest, inasmuch as they have corrupted and endeavoured to destroy true medicine by their avarice and tyranny, and no doubt would have accomplished their ends, had not God raised up honest persons who have strongly and courageously opposed their wicked designs. As for me, I neither like or fear them. If they deprive me of practice, I shall find other for my recompense, and on no account whatever would I accept their friendship. * * * * * * * * Polypharmacy never yet made a good physician; *ad bene medendum, pauca, sed selecta et longo usu probata requirantur remedia, tempore et loco adhibito.*"

There can be no doubt that many of Patin's strictures upon the enormous abuses of drug-giving prevailing at the epoch were well-founded: but he is no less over-bearing and severe towards another class of practitioners, the *Surgeons* and *Barber-surgeons*—the "booted lacqueys" of the physicians, as he contemptuously calls them. Every effort was opposed to all their attempts at increasing their acquirements and extending their sphere of action: and, so many years after Ambrose Paré had cast such lustre upon French surgery, we find the domineering superiority of the physicians rigorously maintained. It is true that certain operations, such as lithotomy, were performed by a special class of persons, who seem to have been held in considerable estimation by the Faculty.

"As to the surgeon-barbers they are never admitted without an approbation, and are examined in our presence. They are permitted to practise nothing but surgery—no pharmacy whatever; and especially neither purgative or anodyne may be given *sine prescriptione medici.*"

A species of tribute seems annually to have been paid as an acknowledgment of the superiority of the physicians.

"After the submission which the surgeons are accustomed to make to the Faculty, they pay to the Dean 100 *sous tournois* for annual quittance, which has long been a mark of their subjection to the Faculty. Besides this, each Master, on the day of his election, pays, from gratitude to his good mother the Faculty, to the Dean four livres for his reception, which we take care to make them pay, if they themselves forget this quittance."

Nothing could be more reprehensible than the conduct of Patin and his colleagues towards the surgeons (so called as distinguished from the mere barber-surgeons) who, from having received an academical education, were termed "surgeons of the long robe," and who were anxious to incorporate themselves into an independent college, with appropriate costume, and power to grant degrees. All attempts of this kind were fiercely opposed by the Faculty, and after long litigation, a decree confirmatory of the predominance of this body was obtained from the Parliament so late as 1660 and 1676. "Surgery was degraded in 1660," says Louis, in his History of the Royal Academy of Surgery, "and yet, when the Academy of Sciences was established in 1666, the surgeons were admitted therein, and maintained a distinguished rank among the illustrious men whom the government presented to the nation as the chosen among her philosophers." M. Parise furnishes us with a copy of the oath the surgeons and apothecaries were compelled to take before the Faculty of Medicine, the latter only being admitted when the former had retired. The surgeon's oath runs thus :—

"You swear you will obey the Dean of the Faculty in all things honest and permissible; as well as pay the same honour and respect to the doctors of the Faculty as scholars owe to their masters. 2. That you will not divulge the secrets of the Faculty, supposing that you know them, and, on the contrary, you will reveal to it whatever you may learn is being contrived against its interests. 3. That you will proceed determinedly against all those who practice medicine illegally, that is, those who have not been approved by the Faculty, and that you will aid with all your might all the proceedings it may take against them. 4. That you will not execute the orders of any physician unless he be a doctor of the Faculty or approved by it. 5. That you will administer no purgative or alterative or cordial medicine; and that you will interfere only with what concerns the manual operations of surgery."

It may be easily imagined that the surgeons made every effort to get so iniquitous a decree repealed, which we believe was only accomplished when the various institutions were re-modelled during the Revolution. Patin notices these attempts with his usual vituperations.

"Most of our surgeons are great rascals, *putidissimi nebulones, iniquissimi ardeliones*. By means of the king's first barber, whom they would much prefer having at their head to being subjected to our Faculty, which has brought them up, preserved them, and maintained them to the present time, they obtained a respite of the execution of the decree. Next day they began defying us, and replaced on their gate the word '*Collegium*,' which they had effaced. Three days afterwards our Dean procured another decree, which commanded them to discuss the matter no farther, but to obey the original one. The King even said he would not interfere in the matter. It is a race of vipers that is constantly rebelling against justice and honesty."

Patin's animosities were far from being confined to professional delinquents. Cardinal Mazarin, the monks and priests, especially the Jesuits, the popes, and their agents, all come in for a share of his wrath and sarcasm. And when we contemplate the corrupt condition of the ecclesiastics, the profligacy of the higher ranks, and the miseries, oppressions, and exactions to which the masses of the people were subjected, as abundantly set forth in these letters, we can well excuse the strong expressions of this indignant exposer of abuses, which, allowed to continue and accumulate for another century, at last gave rise to the terrible revolutionary tempest that shook Europe to its very centre. To this part of his letters, however, we can only direct the attention of those of our readers desirous of studying the facts of history rather than the opinions of historians, as containing for them a rich fund of information.

The student of literary history will also derive much gratification in the perusal of these volumes from the very numerous and often valuable bibliographical notices they contain. Patin was an erudite scholar, and a man of taste and critical acumen. He accumulated a valuable library of some ten thousand volumes, and was ever upon the look out to procure, at any expense, the new works of note as they appeared: and many are the interesting comments we meet with upon Salmasius, Riolan, Erasmus, Gassendi, Grotius, Scaliger, Hoffman, and a host of others, not to mention the ancients, of whom he was a passionate admirer, and frequently a most pertinent quoter. He also much occupied himself in accumulating the best theses he could meet with, and his letters are constantly referring to the means of procuring these and other new publications, which it would seem were at that period more often printed in Holland and England than in France, a rigid censorship of the press being maintained by the Jesuits in this last country. The lovingness and respect with which he speaks of his favourite writers, and his anxiety for new productions from the pens of those still alive, have something even affecting in them. Never is Patin so happy as when in his study, dipping into his Galen or Virgil, or devouring the last new work of Riolan or Salmasius; and the ire which the bold practice of some "stibial" doctor, or the pretensions of some unfortunate chirurgeon had recently excited is exchanged for the enjoyment and grateful expressions of the literary enthusiast. "My little library," he exclaims, "is the light of my eyes and the solace of my labours." "I hold myself to be happier there with my books and a little leisure, than is Mazarin with all his riches and his cares." His range of reading was large, embracing the whole field of the belles-lettres, perusal of works relating to which he termed his *debaucheries*, as compared with the sterner reading of the medical classics he so much admired.

Although a severe censurer of the conduct of the great, and manifesting on many occasions very democratic opinions, Patin was always a loyal subject, and put great faith in the good disposition of the young king proving a corrective for the evils of the state. The decapitation of Charles I. by the English seems much to have startled him, and he always speaks of us as a very ferocious people, as we were doubtless considered in those days on the Continent, traces of such feeling even yet remaining there. He the more cordially disliked us from the fact of one of his most favourite writers and intimate friend, Salmasius, having written so forcibly in condemnation of the proceedings of Cromwell and his followers.

"As for the English, if you except a small number of honest persons, I wish them as much harm as they have done to their king. It is a haughty, proud, and malignant nation, '*quæque πατροπαραδοτον habet odisse Gallos*,' as Scaliger somewhere says in his beautiful letters. * * * * The English are *crudeles et feroces*. Theodore Macille said they were a species of man *de genere lupino*; as the Spaniards and Italians were of the nature of the fox, *callidi, versipelles et astuti*. The Jesuits are hermaphrodite, wicked as the English, and cunning as the Italians. * * * * * The Queen of England arrived to-day at Calais, where her son the Duke of York came to meet her. The executions are going on in London, where there have been already ten hung. The last were two colonels, who were ordered by the Parliament to see execution done on the late King. All these criminals are strange people, who will repent nothing either of the fact or the death. They are martyrs of the State and of the times. It seems to me they are quite infatuated. I think this only occurs in that nation which seems to differ from all others. They are so cruel, ferocious, and blood-thirsty, as to be nearly fatuous."

It may be believed that one so jealous of the dignity and privileges of the Faculty as Patin was not indifferent to the highest honours it had in its gift; and accordingly we find him frequently alluding to his two successive elections as Dean of the body with evident satisfaction; although, by reason of the dissensions excited among its members by the advocacy of the claims of antimony, matters did not always run so smooth as might be desired. In fact, the Dean, as M. Parise observes, "was a dictator at the head of a republic difficult enough to govern." The following is Patin's description of his functions, and the somewhat complex mode of election.

"He is master of the bachelors, and regulates the discipline of the schools. He keeps our registries, which are 200 years old, as well as the seals of the Faculty. He receives our revenue, and renders us an account of it. Every thesis requires his signature and approbation. He arranges the order of precedence of the doctors, and assembles the Faculty only when he pleases. He, with the four examiners, conducts rigorous examinations, which last for a week, and he is one of the three deans of the University. It is a laborious office, one of much honour, but harassing in its duties. He conducts the actions brought by the Faculty, and even pleads before the Advocate-general. It is a very honorable but a most laborious charge, which a worthy man ought to feel very happy in escaping, satisfied in being thought worthy of filling it by being elected to it. This is the mode of the election. All the Faculty being present, the Dean who is going out of office thanks the company for the honour they have done him, and desires they will elect another in his place. The names of all the doctors present are written on so many tickets and laid on the table. The first half of these are taken and placed in a hat, and are called the great bench. We are now 112 in number, so that the first 56 are placed in the hat. When these tickets have been well shaken by the oldest of our company, who is M. Riolan, the Dean quitting office draws three names one after the other, and draws two others from the little bench. None of these five doctors can be the Dean on that day, but they are the electors, who, after having taken an oath of fidelity, are shut up in the chapel, where they choose the names of three of the assembled doctors whom they believe worthy of the office, two from the great bench and one from the little one. These names are put in the hat by the senior doctor, and the Dean, placing therein his hand wide open, draws one who is to be the future Dean. I have been several times an elector, and have even been placed in the hat three times, and have remained there, and I shall not be sorry to do so whenever I am put there, for I have no leisure for such a charge *sortes in urnam mittuntur, sed temperantur a Domino.* All these ceremonies are very ancient, and are religiously observed out of respect to their antiquity."

Patin, however, at last attained the honor, and his active disposition found time to creditably fulfil the duties, some of which, such as presiding at the humiliating admission of the surgeons and apothecaries, pursuing illegitimate practitioners, and holding disputations on the theses, were eminently congenial to his tastes. The disputation of the theses was one especially so; and he constantly refers in his letter to the collection of those he and his friends are making, negotiating exchanges, purchases, &c., or liberally presenting them. "I can give you several," he says in one letter, "seeing I have always collected as many as possible, so that I have here more than 700 in good order, besides a great number of duplicates." Some of these productions were of great merit, and had a large circulation, but even some of the most approved in that scholastic and disputatious period would seem oftentimes insignificant to our more practical age. But, although the authority of the ancients and the schools was still predominant and indisputable in the Faculty, the means of correcting their errors were already in operation: for not only had the propriety of administering the various new remedies, cinchona, antimony, mercury, and opium, been admitted by a majority of the doctors; but the study of human anatomy was pursued with zeal, though not with much regularity. Patin in his letters repeatedly gives us accounts of the postmortem examinations he had been present at; and frequently interrupted his courses of lectures in order that his pupils might have the opportunity of witnessing the dissection of criminals—opportunities, indeed, from the dreadful frequency with which capital punishment was at that period inflicted, of no uncommon occurrence.

A curious and somewhat undignified custom prevailed with the Faculty, by which all the candidates, received or rejected, were required to provide a handsome entertainment for their examiners and a number of the Faculty besides. "We formed three tables, at each of which were from twelve to thirteen of us. I never saw such rejoicing, such laughter, and such good cheer." Imagine the rejected of the College of Physicians or Surgeons thus contributing to the gladdening the hearts of their examiners! The custom prevailed till recent times, for the celebrated Pinel, although received as physician at Montpellier, having been rejected at Paris, was still obliged to pay his share of the banquet. As Patin often mentions that many of the Doctors (especially those who prescribed antimony) were much addicted to deep potations, we can easily imagine that such scenes did not always reflect credit on the learned body.

The Paris Faculty seems always to have been on terms of bitter rivalry with the other establishments for medical education, especially the Faculty of Montpellier; and, Patin again and again accuses them of disposing (an abuse prevalent amongst some "learned" universities of our own times) of their degrees for mere pecuniary considerations.

"If the physicians of Montpellier are badly paid by their patients, they recompense themselves by giving licenses for whoever will pay for them: *modo fiat nummis præsentibus.* It is an abuse I am astonished at, but have no means of preventing. * * * * * * The little universities *manifeste peccant in publica commoda;* they reject no one. If the young doctor is not received cheaply at one place he goes to another: and this is why the physicians of Rheims are now pleading against those of Angers; seeing that they sell their degrees cheaper, after a slight examination, in a little time, and if it is desired even without theses. Really if some remedy be not found for this disorder, we shall have more physicians in France than there are apples in Normandy or priests in Spain and Italy. Without exaggerating their ignorance, which is in truth extreme, shameful, and dangerous, they will not even study or have any books, it being enough for them *si habeant in manibus diplomata academica, etiam vili ære redempta,* and if they are the cousins or neighbours of some surgeon or apothecary. I have seen some of them who have even forged their diplomas. They repair to their native country, village, or small town, with scarcely a *Perdulcis* or a *Fernel* about them, or not understanding them, pretending however to do so, just as if they had the *jus vitæ et necis.*"

It must not be believed from the scraps of Latin with which Patin, after the manner of his time, delights to interlard his letters, that he was any pedantic smatterer. On the contrary, those of his letters written entirely in that language, and the numerous and apt quotations from the poets in the present collection, prove him to have been an elegant scholar; and indeed it is surprising, amidst his numerous professional occupations, that he could have acquired so intimate an acquaintance with the literature of antiquity and of his own times.

We have space only for one other of the numerous extracts we had marked, and this relates to the pathological indications of *red hair*.

"The disease you have taken the trouble to describe to me is somewhat of a gouty nature. I know the patient whose complexion is delicate. His father, who had black hair, died of a pulmonary catarrh, and his mother of an inflammation of the lungs. She was the most quarrelsome and choleric woman in existence, and more than that, she had very red hair. Now, it is constantly observed that, inflammation of the lungs is fatal in the red-haired. I was called in consultation with the late M. De la Vigne upon the case of Collier, Secretary to the King, who was 75 years of age, and suffering from inflammation of the lungs. As he had red hair, I at once predicted a fatal issue. M. De la Vigne inquired where I had learned this prognosis. I replied that I had always observed it to be a very true one, besides which, I had heard it from M. N. Piètre, who had it from his brother, the great Simon Piètre, and that its explanation was, that red-haired persons abounded in an acrid and malignant serosity. He replied that he had always observed the same thing, and I have since read of it in the *Ephemerides* of Baillou."

In concluding our notice of these interesting Letters we feel, as we feared on commencing it, that we have been enabled to convey only a very inadequate idea of them to our readers. But at least they will be aware of the existence of so remarkable a book; and will do well to put themselves in possession of it. Numbers of topics are embraced within the scope of the correspondence, relating to the politics, the general literature, the manners, and the gossip of the day, to which our limits and the objects of this Journal alike prevent our alluding. To M. Reviellé-Parise the medical profession in France owe much for recalling their attention to these interesting letters and their once celebrated author.* It was a work of love and of labour, and we suppose no where could an editor have been found more fitting for the task. Himself an elegant writer and acute critic, evidently an admirer, though not a blind one, of the erudition of a bye-gone age; well versed in the literary history of our profession, and indeed in general literature; a firm upholder of the true dignity and rights of medicine when pursued as a liberal profession and not for mere lucre; but a stern denouncer of all charlatanerie, professional and non-professional; he is fully enabled to enter into the spirit and views of his author, to indicate at once his merits and his failings. This he has carefully and faithfully done in a series of "scientific, historical, philosophical, and literary" footnotes, which add much to the value of the book, by explaining some obscurities, and indicating comparisons between the state of medicine as it was and as it is—not invariably, however, in favour of the latter. The preparation of these, and the rectifying the numerous errors of the spurious editions of Patin's letters, by a comparison with the difficultly-decipherable originals, must have been a work requiring untiring patience.

---

* Alas! how fugitive is fame, unless built upon some solid undertaking which may descend to posterity. The reputation of Guy Patin during his life-time was European, and shortly after his decease several collections of his letters were published and eagerly read; and yet, in less than a couple of centuries afterwards, several well-informed persons are found calling at the publisher's, upon hearing of the new edition, to inquire who is this physician and where is his abode!

